# Deriving Large-Scale Coastal Bathymetry from Sentinel-2 Images Using an HIGH-Performance Cluster: A Case Study Covering North Africa’s Coastal Zone

**DOI:** 10.3390/s21217006

**Published:** 2021-10-22

**Authors:** Mohamed Wassim Baba, Gregoire Thoumyre, Erwin W. J. Bergsma, Christopher J. Daly, Rafael Almar

**Affiliations:** 1 CNRS-LEGOS, UMR-5566, 14 Avenue Edouard Belin, 31400 Toulouse, France; gregoire.thoumyre@legos.obs-mip.fr; 2 Center for Remote Sensing Application (CRSA), University Mohammed VI Polytechnic (UM6P), Benguerir 43150, Morocco; 3 French Space Agency (CNES), 31400 Toulouse, France; erwin.bergsma@cnes.fr; 4 IRD-LEGOS, UMR-5566, 14 Avenue Edouard Belin, 31400 Toulouse, France; dalyjchris@gmail.com (C.J.D.); rafael.almar@ird.fr (R.A.)

**Keywords:** bathymetry, Sentinel-2, remote sensing, North Africa, HPC

## Abstract

Coasts are areas of vitality because they host numerous activities worldwide. Despite their major importance, the knowledge of the main characteristics of the majority of coastal areas (e.g., coastal bathymetry) is still very limited. This is mainly due to the scarcity and lack of accurate measurements or observations, and the sparsity of coastal waters. Moreover, the high cost of performing observations with conventional methods does not allow expansion of the monitoring chain in different coastal areas. In this study, we suggest that the advent of remote sensing data (e.g., Sentinel 2A/B) and high performance computing could open a new perspective to overcome the lack of coastal observations. Indeed, previous research has shown that it is possible to derive large-scale coastal bathymetry from S-2 images. The large S-2 coverage, however, leads to a high computational cost when post-processing the images. Thus, we develop a methodology implemented on a High-Performance cluster (HPC) to derive the bathymetry from S-2 over the globe. In this paper, we describe the conceptualization and implementation of this methodology. Moreover, we will give a general overview of the generated bathymetry map for NA compared with the reference GEBCO global bathymetric product. Finally, we will highlight some hotspots by looking closely to their outputs.

## 1. Introduction

Coastal morphology plays vital role in the global environment. It could be considered as a barrier between land and sea. Coastal regions with shallow water are subject to permanent changes. This impacts their vulnerability to coastal flooding. Moreover, it also can harm economies—e.g., the Netherlands invests on average EUR 35,000,000.00 per year to nourish the coast [1]. Moreover, the depth and shapes of the underwater bathymetry are important for the maritime sectors [2]. Bathymetric maps are also used in biological oceanography, because the depth of the sea is linked to biological characteristics of marine ecosystems. Therefore, generating high-resolution bathymetry maps is an added value to several domains. Despite this major importance, it is very challenging to create bathymetric maps with both accurate spatial resolution and large spatial coverage. It is both time-consuming and very expensive [3]. In recent decades, single-beam echosounding and many other technologies such as multi-beam echosounding, Light Detection and Ranging (LIDAR), have been used to produce the bathymetry of coastal regions at different resolutions and accuracy [3,4,5]. Classical studies of surveying coastal regions, generally use acoustical techniques which consist of measuring the distance between the device and the bottom of the sea. This technique offers a good accuracy but it is very slow and covers very limited areas. An alternative that can scan a wide area with a good spatial resolution is LIDAR. It uses infrared and green laser transmitter and post-flight data processing techniques to generate survey depths with high accuracy [6,7]. However, this technique is more expensive, especially when the study area is large. To overcome technical and economical issues of in situ observations, the new generation of spaceborne optical remote-sensing sensors could be a good alternative. It is characterized by high spatial resolution and regular revisit time (varying from a few days to 16 days) while covering the entire globe [8]. Several studies are currently moving in this direction, and try to use remote sensing data to retrieve the water depth in coastal regions. Hamylton et al. [9] compared two approaches to estimate the bathymetry at 5 m of resolution. This study was established at both the Lizard Island and Sykes Reef sites, by using WorldView-2 images. Chybicki [10] demonstrated that an inversion of Sentinel 2A/B radiance is useful to derive an estimation of the shore bathymetry (for areas with a depth lesser than 18 m in South Baltic coastline. Other researchers used similar method to derive the bathymetry such as [11,12]. This method is efficient for nearshore areas but has a principal limitation of this method is the water turbidity which degrades the quality of the results. Thus, the objective of this article is to present a large-scale implementing an High Performance Cluster (HPC) methodology to derive the bathymetry from high spatial resolution images (of type Sentinel 2A/B) from a regional, continental to global scale. We present an approach that processes many images simultaneously with a computation time around 1.5 h per image per CPU (central processing unit). At first stage, we describe physical laws that control the estimation of the water depth followed by a detailed HPC-workflow to implement the bathymetry derivation-code. In the results section we present a study case applied in North African’s coasts. Detailed description of computational time and memory while running the code over the tiles will be given. Finally, in discussion section we discuss the limitations of the methodology, and present a recommendation to run it over the world with specific IT resources.

## 2. Study Area and Data Source

### 2.1. Study Area

**Figure 1:**
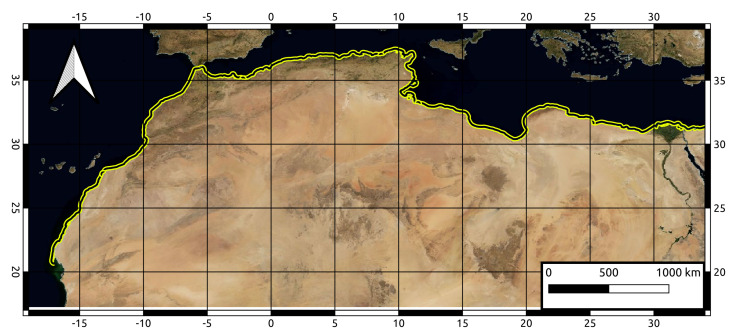
Contextual map of our study area: coasts of North Africa are represented by the yellow buffer. Reference coordinate system used: World Geodetic System (WGS84).

The arid coasts of North Africa, extending over more 6000 km from Morocco Atlantic Coast to the Nile Delta (Figure 1), are undergoing pronounced shoreline retreats and coastal flooding that are reported as a consequence of the ongoing sea level rise resulting from global warming. The coastal zone plays an important role in the economic development of this region [13]. Indeed, more than 60% of the population live in coastal cities, and 90% of the country’s industries are based along the coast [14,15]. Of particular interest are the abnormal shoreline dynamics for deltaic and sandy beaches, which are severely impacted by abrupt decadal variabilities in both climatic and anthropogenic drivers resulting in their increased vulnerability to disturbances from coastal hazards. Unfortunately, the evolution, distribution and impacts of these drivers remain largely unquantified, let alone understood, for these extensive arid coasts that harbor the major portion of North Africa’s population as well as unique and fragile marine ecosystems.

### 2.2. Sentinel 2A/B Images Retrieval

Sentinel 2A and 2B are two twin polar-orbiting satellites launched in 2015 and 2017, respectively, as part of the European Copernicus program [16]. They are designed for the operational monitoring of atmosphere, land and ocean. With a wide-swath and a multispectral imager (MSI) with 13 spectral bands (from 443 nm to 2190 nm), a high spatial resolution imagery varying from 10 m for the majority of the bands covering the visible, and very near infrared (VNIR), to 60 m for the short wave infrared (SWIR). In addition to this variety of bands and the high spatial resolution, Sentinel 2A/B has a temporal resolution reaching a maximum of 5 days (more you are far than equator less the temporal resolution [17]). They allow the observation and monitoring of land, atmosphere and ocean every 5 days.

In this study, 287 Sentinel 2A/B tiles cover the North Africa coastal zone (Figure 1) were used. First, we queried all available Sentinel 2A/B (S2-L1C) images between January 2015 and January 2020 in the PEPS collection of the Theia Land data center. For the analysis in this work we limit the computations to ten scenes of cloud-free Sentinel 2A/B images during a 5 year span from 2015 to 2020. A sensitivity analysis in the original method article [18] and for West-African hotspots [19], shows that the wave power (as a function of wave height and period) as well as directional spread of the wave field affect depth estimation. Generally, the more powerful, narrow-spread wave fields allow for a more accurate estimation of the water depth.

Considering this, 10 cloud free images with most powerful waves are selected in this study. Since we are working on 287 tiles and in each tile there are 10 images, we integrated 2870 images in the HPC. If our area (the selected image) is totally covered by water, it has an execution time of 72 core-hours (36 CPUs used for two hours). If the image has a terrestrial part this time decreases. This is why we fixed walltime for running the Portable Batch System (PBS) in 2 h.

#### Global Water Mask

Since it is not our objective to estimate water depths on lands, the land is filtered from the image by using a land-mask. The set of routines is fed by a water mask which not only prohibits depth estimation at land but it is also used to compute the distance to the shore that is used in adaptive tile-sizes during the computation. Here we use an existing global water mask with an initial resolution of 300 m. It was generated by European Space Agency Climate Change by combining SAR images and recent Landsat-derived products. The water mask was re-sampled to a resolution of 500 m in order to be compatible with our outputs: water depths are estimated on 500 m resolution.

## 3. Methods

### 3.1. Physical Description

Ocean waves, when they are about to arrive in the coastal zone, are free moving waves. Only in intermediate $h_{int}=\frac{L}{2}$ to shallow water $h_{sh}=\frac{L}{20}$, the propagation of these waves is limited by, among other physical processes, the water depth. As the water depth reduces towards shore, waves increasingly “feel” the bottom by increasing bottom-friction until the waves eventually break close to shore. Depth domination of the wave propagation can be described with through a mathematical relationship; the dispersion relation for free surface waves.

(1) $$c^{2}=\frac{g}{k}\tanh\left(kh\right)\Leftrightarrow h=\frac{\tanh^{-1}\left(\frac{c^{2}k}{g}\right)}{k}$$

To solve (1), one is after a pair of wave celerity (*c*), wave length (*L*) or number (*k*) and wave period (*T*) or frequency (ω). Here we use the approach following Bergsma et al. [18] to find wave phase shifts (leading to celerity *c*) per wave number (*k*) in different detector-bands using a Radon-Transform based Fourier slicing techniques. The great benefit of this Radon-Transform based technique is the limited dependence on image resolution to estimate wave propagation while maintaining computation performance.

### 3.2. Numerical Implementation and Regional Application

#### 3.2.1. IT Workflow

**Figure 2:**
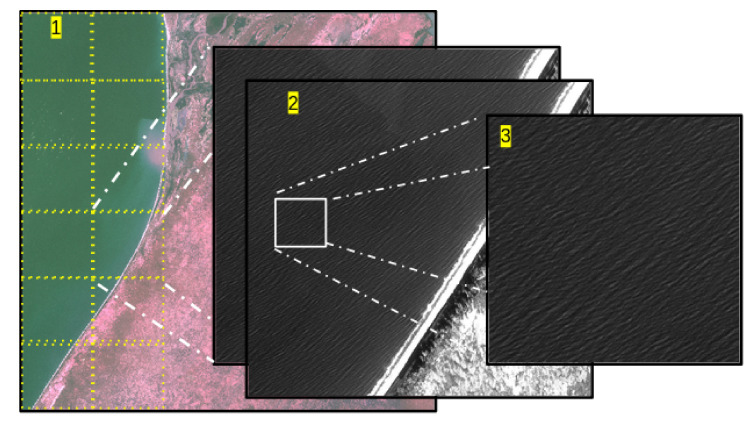
(1). Description of the manner by which the slicing is performed depending on the number of CPUs. (2). Creating a sub-window over the point of interest. (3). The sub-window where the different variables (e.g., celerity and depth) are computed.

To minimize the execution time, each Sentinel 2A/B image is split into 36 subsets (6 × 6). This ensure that all the available CPUs are used at the same time. Thereafter, we move a sub-window over the pixels that we want to process (depending on the output resolution). In this sub-window the physical equations described in Section 3.1. The approach of this decomposition is illustrated in Figure 2. The size of each sub-window depends primarily on the distance to the shore. It’s equation is given as follows:

(2) $$\kappa =\min\left(2.5,{\left[\frac{\log_{10}\left(D_{shore}\right)}{2}\right]}^{2}\right)$$

(3) $$W_{xs}=\kappa \times L_{win}$$

where, κ is the factor regulating the size of the window. $D_{shore}$ is the distance to the shore and $W_{xs}$ represents the window length (The size of the window is $W_{xs}\times W_{xs}$. This widow is applied for each study bands. $L_{win}$ is constant fixed in 200 m in this study.

Once the pixel is surrounded by the window, a Radon Transform (RT) is applied as described in [18]:

(4) $$R_{sub_{I}}=\mathrm{radon}\left(sub_{I}\right)$$

$R_{sub_{I}}$ represents the sinogram calculated over the sub-window domain. This sinogram allows to extract the main direction of the wave by taking the maximum variance or standard deviation over all directions. Once the direction is found, a fast-Fourier transform DFT is performed over beam with the selected angle to obtain the phase per band (ϕ) (following the methodology of [18]). Using two images with a slight δ(t), one can compute the phase shift Φ and find celerity (*c*) by linking λ, Φ using (5)

(5) $$c=\frac{\Delta \Phi }{2\pi \lambda \delta (t)}$$

where, δ(t) represents the difference time in seconds (s) between the acquisition detector bands. Here only the 10-m resolution bands are used, but possible Radon-augmentation enables the use of all bands [18].

**Figure 3:**
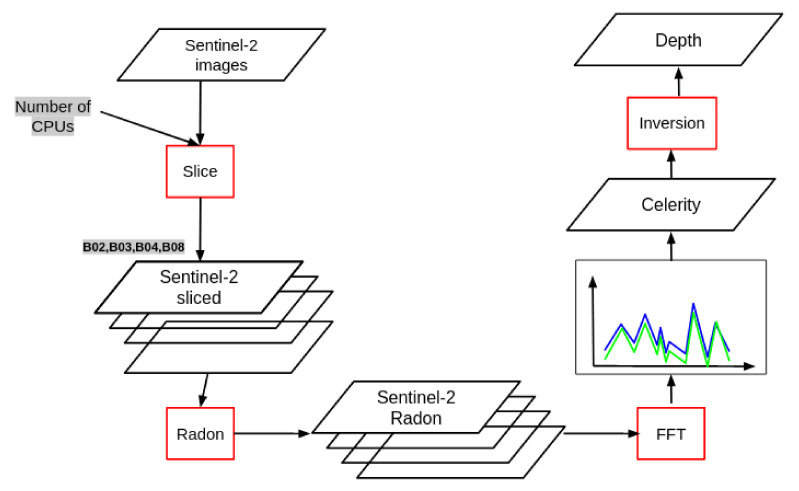
Workflow for Sentinel 2A/B process, from the acquisition to retrieving the bathymetry.

The extract the depth (*D*) a numerical implementation of Equation (1) is implemented. A graphical description of the algorithm that summarizes the workflow is given in Figure 3. The IT implementation of the physical code is presented in Section 3.1.

#### 3.2.2. HPC Implementation

A High Performance Computing (HPC) techniques refers to a supercomputer with a high level of performance as compared to a general-purpose computer. The basic elements of HPC techniques consists of node, queue, and a job. A node is a single physical or logical computer with one or more processors. Queuing systems are responsible for managing job requests which are shell generally scripts submitted by users. A job is a collection of instructions that a user initiates. Each job reserves specific resources in term of Random-access memory (RAM) and CPUs. To sum up, the computations are performed by the cluster, by submitting a job request to a specific batch queue. The scheduler will assign your job to a compute node in the order determined by the policy on that queue and the availability of an idle compute node.

**Figure 4:**
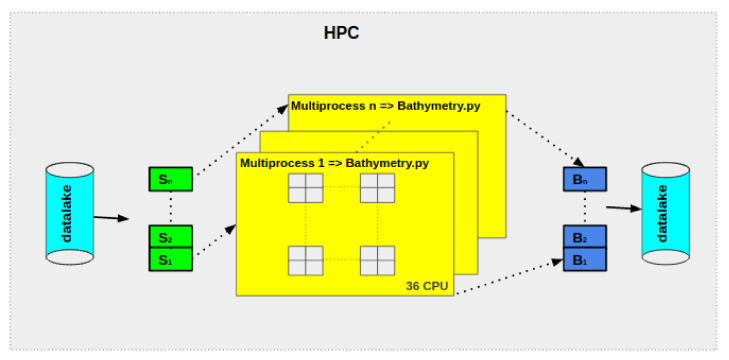
Schematic overview of a HPC architecture. From the right to the left: loading of the data from the datalake Sentinel-1, 2, ..., n. Then in each node (the yellow box) the image is split into 36 sub-images that are treated in parallel. Each output is merged and then saved in the datalake.

In our case, the different simulations were established in the CNES-HPC cluster. All available cores are allocated with 36 Central Processing Units (CPU) and 70 Gb of RAM. The data are collected from the datalake; thereafter, each node treat separately one image (Figure 4).

### 3.3. North Africa Coastal Bathymetry Showcase

**Figure 5:**
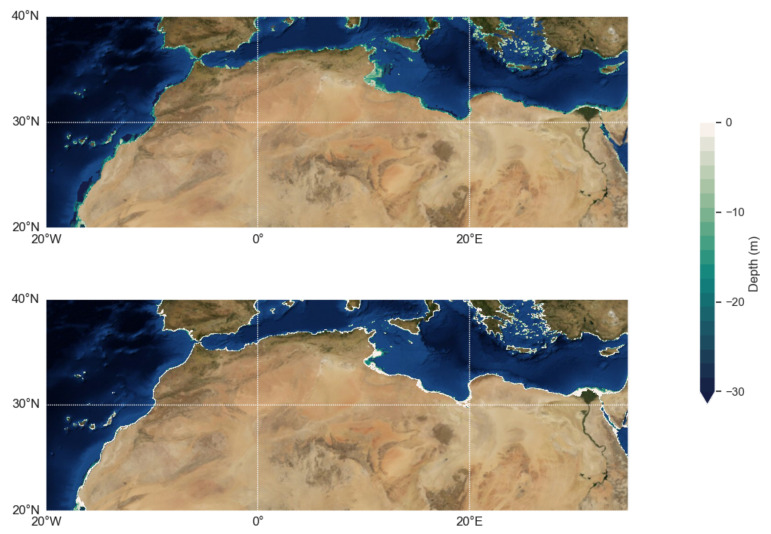
Bathymetry along the North Africa coastline with our S2 shores estimate at the top and the reference GEBCO global product below.

Water depth maps are at 500 m of spatial resolution. Figure 5 shows the result of the computation for the North Africa coastal region. Bathymetry is computed until the 2% of potential estimated depth [17] using ERA5 hindcast (ECMWF). This covers most of the shallow shelves, bays (Gabes bay) and delta (Nile). This potential varies from shallow in the Mediterranean Sea, due to limited fetch to deeper waters along the open Atlantic Coast. S2Shores results show in general a good agreement with GEBCO, the referent global bathymetry product, combining numerous surveys and previous altimetry-based coarse satellite estimations.

However, it was found that GEBCO has substantial issues in coastal and shallow waters, and often generates some bumps due to interpolation of different source data-set and ends up at the shore with a linear interpolation to the shoreline. A more thorough validation from an unrivaled local survey remains invaluable in terms of ground truth and is yet to be done in this area. However, [19] conducted in Senegal a comparison with echo-sounder surveys and our estimated showed an accuracy of meters (RMSE is varying between 2 and 5 m, i.e., 10–20%).

**Figure 6:**
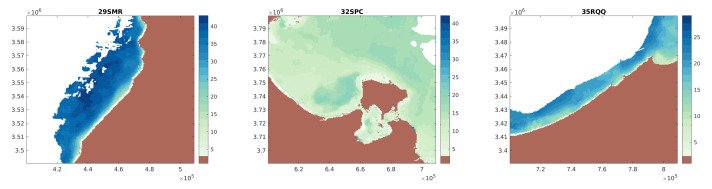
Illustration of S2Shores satellite-derived coastal bathymetry at showcases zones. The the tiling grid IDs for Sentinel 2 are given at the top of each image.

The implemented method allow to represent different types coasts varying from shallow depth to deep depth as shown in Figure 6 for the three different coasts in Morocco; Djerba island, Tunisia; and Egypt. In these cases the maximum depth, or deep water limit is reached around 45 m.

## 4. Conclusions and Way Forward

By positioning itself on a global coverage, S2SHORES covers different coastal zone environments around the world with a sensor resolution of 10 m (Sentinel 2A/B) over a variable band from the shore and down to a depth limit of about 50 m [19]. The approach can be extended to other missions (Pleiades, World-View, SPOT6, etc.) of higher resolution for high precision need at hot-spots [20]. Sentinel 2A/B assets are the free data and the long-term nature of the Sentinel 2A/B program. Indeed, Sentinel-2A and B cover the periods 2015–2022 and 2017–2024, respectively, and their successors Sentinel-2 C and D to ensure the continuity of the mission are already scheduled for the next seven years. We show here that the recent availability of new global high resolution products such as Sentinel 2A/B (ESA) COPERNICUS constellation, combined with the latest methodology of bathymetry retrieval, can be applied globally: offering a new vision with uniform method. The results presented here are a pathway toward new EOS coastal products. Besides the approach employed and the scores, the new possibilities are evident. There is a current strong and increasing demand for such global product, after the decades old supremacy of state-of-the-art global relatively coarse resolution and rather inappropriate at the coast. This for coastal engineering, management and planning, risk forecasting and mitigation abut also scientific advance. The way forward include the optimal use of the regular, and increasing, revisit of these earth observation satellite to monitor coastal changes under climate change. 

## Data Availability

The S2Shores maps are available at CNES datalake. Please contact R.A. (rafael.almar@ird.fr) if you are looking for their use.

## References

[1] Jonkman S.N., Hillen M.M., Nicholls R.J., Kanning W., van Ledden M. (2013). Costs of Adapting Coastal Defences to Sea-Level Rise—New Estimates and Their Implications. J. Coast. Res..

[2] Guenther G.C. (2007). Airborne lidar bathymetry. Digit. Elev. Model Technol. Appl. DEM Users Man..

[3] Irish J.L., White T.E. (1998). Coastal engineering applications of high-resolution lidar bathymetry. Coast. Eng..

[4] Heezen B.C., Tharp M. World Ocean Floor Map. https://www.lib.uchicago.edu/collex/exhibits/marie-tharp-pioneering-oceanographer/1977-world-ocean-floor-map/.

[5] Barton C. (2002). Marie Tharp, oceanographic cartographer, and her contributions to the revolution in the Earth sciences. Geol. Soc. Lond. Spec. Publ..

[6] Guenther G.C., Thomas R.W., LaRocque P.E. (1996). Design considerations for achieving high accuracy with the SHOALS bathymetric lidar system. CIS Selected Papers: Laser Remote Sensing of Natural Waters: From Theory to Practice.

[7] Irish J.L., McClung J., Lillycrop W.J. Airborne Lidar Bathymetry: The SHOALS System. https://trid.trb.org/view/652843.

[8] Cazenave A., Le Cozannet G., Benveniste J., Woodworth P., Champollion N. Monitoring Coastal Zone Changes from Space. https://eos.org/opinions/monitoring-coastal-zone-changes-from-space.

[9] Hamylton S.M., Hedley J.D., Beaman R.J. (2015). Derivation of high-resolution bathymetry from multispectral satellite imagery: A comparison of empirical and optimisation methods through geographical error analysis. Remote Sens..

[10] Chybicki A. (2017). Mapping south baltic near-shore bathymetry using Sentinel-2 observations. Pol. Marit. Res..

[11] Stumpf R.P., Holderied K., Sinclair M. (2003). Determination of water depth with high-resolution satellite imagery over variable bottom types. Limnol. Oceanogr..

[12] Lyons M., Phinn S., Roelfsema C. (2011). Integrating Quickbird multi-spectral satellite and field data: Mapping bathymetry, seagrass cover, seagrass species and change in Moreton Bay, Australia in 2004 and 2007. Remote Sens..

[13] Hzami A., Heggy E., Amrouni O., Mahé G., Maanan M., Abdeljaouad S. (2021). Alarming coastal vulnerability of the deltaic and sandy beaches of North Africa. Sci. Rep..

[14] Snoussi M., Ouchani T., Niazi S. (2008). Vulnerability assessment of the impact of sea-level rise and flooding on the Moroccan coast: The case of the Mediterranean eastern zone. Estuar. Coast. Shelf Sci..

[15] Hakkou M., Maanan M., Belrhaba T., El Khalidi K., El Ouai D., Benmohammadi A. (2018). Multi-decadal assessment of shoreline changes using geospatial tools and automatic computation in Kenitra coast, Morocco. Ocean Coast. Manag..

[16] ESA Sentinel-2 Delivers First Images. https://www.esa.int/Applications/Observing_the_Earth/Copernicus/Sentinel-2/Sentinel-2_delivers_first_images.

[17] Bergsma E.W.J., Almar R. (2020). Coastal coverage of ESA’ Sentinel 2 mission. Adv. Space Res..

[18] Bergsma E.W.J., Almar R., Maisongrande P. (2019). Radon-Augmented Sentinel-2 Satellite Imagery to Derive Wave-Patterns and Regional Bathymetry. Remote Sens..

[19] Daly C.J., Baba W., Bergsma E., Almar R., Garlan T. The New Era of Regional Coastal Bathymetry from Space: A Showcase for West Africa using Sentinel-2 Imagery. https://eartharxiv.org/repository/view/187/.

[20] Almar R., Bergsma E.W., Maisongrande P., de Almeida L.P.M. (2019). Wave-derived coastal bathymetry from satellite video imagery: A showcase with pleiades persistent mode. Remote Sens. Environ..

